# Formulation and Evaluation of Spray-Dried Reconstituted Flaxseed Oil-in-Water Emulsions Based on Flaxseed Oil Cake Extract as Emulsifying and Stabilizing Agent

**DOI:** 10.3390/foods10020256

**Published:** 2021-01-26

**Authors:** Emilia Drozłowska, Artur Bartkowiak, Paulina Trocer, Mateusz Kostek, Alicja Tarnowiecka-Kuca, Łukasz Łopusiewicz

**Affiliations:** Center of Bioimmobilisation and Innovative Packaging Materials, Faculty of Food Sciences and Fisheries, West Pomeranian University of Technology Szczecin, Janickiego 35, 71-270 Szczecin, Poland; Artur-Bartkowiak@zut.edu.pl (A.B.); p.trocer@gmail.com (P.T.); mkosa9406@gmail.com (M.K.); alicja.tarnowiecka-kuca@zut.edu.pl (A.T.-K.); lukasz.lopusiewicz@zut.edu.pl (Ł.Ł.)

**Keywords:** flaxseed oil cake, flaxseed oil, spray drying, emulsions, stability, reconstitution

## Abstract

Spray drying of emulsions is a promising way of increasing their durability, offering the possibility of reconstitution, with the addition of water. The present study aimed to examine the properties of flaxseed oil cake extract (FOCE) as an emulsifying and stabilizing agent for spray-dried reconstituted oil-in-water emulsions. Maltodextrin: starch: flaxseed oil emulsions with FOCE or distilled water as liquid phases, and 10% and 20% of oil were spray-dried at 180 °C. The solubility, flowability, cohesiveness, bulk, and tapped densities of the spray-dried powders were analyzed. Additionally, the characteristics of initial and reconstituted emulsions, such as stability, creaming index, color, particle size, and rheological properties were evaluated. Results showed that FOCE could be an adequate emulsifier for spray-dried emulsions with a high oil content providing high stability after reconstitution, when compared to emulsions based only on maltodextrin–starch wall material with water as the liquid phase. This study showed an encouraging way for producing natural and plant-based spray-dried oil-loaded emulsions for food applications.

## 1. Introduction

Spray drying is one of the most approachable techniques of microencapsulation, which is prized, simple, and flexible because many parameters (such as inlet and outlet temperature, nozzle size, etc.) could be adjusted. Microencapsulation is described as coating individual particles or droplets with a continuous phase, to obtain capsules in a micrometer to millimeter in size [1]. Spray drying is defined as transformation of a feed from liquid state to a powder by spraying into a hot drying gas [2]. Spray drying process conditions should be well chosen because they affect the physical properties of the powders, obtained over the experiment. For example, process yield, moisture content, particles size, bulk density, color, solubility, and ability for reconstitution. Due to the notable benefits, the spray drying technology finds wide applications in various sectors, such as food, chemical, pharmaceutical, nutraceutical, cosmetic, and biomedical industries.

The fast-growing demand for plant-based foods and food additives, and the current rapid lifestyles resulted in changes of dietary habits that have notably impacted on the growth of interest in powdered food and spray drying technology. Numerous food ingredients and food mixtures could be encapsulated, such as flavors, oils, lipids, emulsions, acidulants, antioxidants, vitamins, minerals, sweeteners, preservatives, colorants, proteins, peptides, and probiotics [3]. The emulsions are spray-dried to extend the period of stability and durability of vegetable and animal oils, preventing against oxidation caused by deteriorating factors (such as humidity, light, and temperature) and destabilization. Destabilization can be the result of a variety of physicochemical mechanisms, such as gravitational separation, flocculation, coalescence, and Ostwald ripening [4]. An emulsion is a thermodynamically unstable complex system. To increase the stability of emulsions, various emulsifiers are applied [5]. In case of the emulsions spray drying process, one of the pivotal factors is the type of wall material. It embraces the core material to protect valuable and bioactive compounds, such as polyunsaturated fatty acids (PUFA) or polyphenols. Moreover, the type of wall material influences flowability, bulk and tapped densities, powder solubility, and their redispersion ability. The production of oil-in-water emulsions is necessary to disperse the PUFA-rich oils in an aqueous phase, to be fed into the spray-drying equipment [6]. Encapsulated emulsions with valuable oils, such a flaxseed oil, could be exploited to fill the ω-3 fatty acids gap in vegetarian diet. The food industries are constantly exploring various raw materials, new technologies, and processes, to evolve innovative functional products. Animal-derived ingredients are widely exploited, although they are considered less environment-friendly, as compared to plant-based ingredients [5]. Additionally, the management of the oil industry by-products that meet the criteria of “zero waste” economy is very important, because a substantial quantities of press cakes and residues are available. Based on the reports of the U.S. Department of Agriculture (USDA), the world production of oilseeds in 2018/2019 was 600.47 million metric tons [7]. According to Ancuța and Sonia, the utilization of oil cakes can be a sustainable way to reduce waste production, as well as contribute to the development of new, cheap, and nutrient-rich products [8].

The aim of emulsifiers addition (such as proteins, polysaccharides, etc.) to emulsion systems is to form interfacial surfaces, due to their capability to adsorb onto oil–water interfaces [9,10]. The emulsifying properties of flaxseed proteins (FP) were already reported [5,11,12,13]. Other essential constituents of the flaxseed are polysaccharides—called flaxseed gum (FG). This gum is also used in emulsion preparation in order to enhance the emulsion stability [5,14]. The mechanism of emulsion stabilization by flaxseed gum is based on increasing the viscosity and decreasing the interfacial tension [15]. The valorization of FOCE (flaxseed oil cake extract, which is a mixed liquid matrix of flaxseed protein and flaxseed gum) in terms of food science is not yet a deeply explored issue. However, there are reports about its technological applications, including stabilization of emulsion systems. Available reports indicate that this valuable agro-industrial by-product fits the idea of circular economy and “zero waste” trends [5,11,12]. In previous works it was demonstrated that FOCE can be applied as an emulsifying agent for stable emulsions with flaxseed oil, due to strong FG and FP synergistic oil binding and water holding abilities [5]. Moreover, it was reported that the spray drying process has a positive influence on the emulsifying properties of FOCE-based spray-dried powders [12].

However, there are no studies available concerning the application of spray-drying FOCE-based emulsions and evaluation of their stability after reconstitution. In this study, our aim was to use the aqueous extract from flaxseed oil cake to obtain spray-dried powders from emulsions, with a 10% and 20% content of FO. Additionally, we also examined their stability after reconstitution.

## 2. Materials and Methods

### 2.1. Materials

The residue from cold pressing of flaxseed oil—flaxseed oil cake (FOC) was donated by the company ACS Sp. z o.o. (Bydgoszcz, Poland). Starch Capsul^®^ (National Starch & Chemical, Bridgewater, Westport, CT, USA) and maltodextrin (PEPEES S.A., Łomża, Poland) were also used. Flaxseed oil (FO) was purchased from Olandia (Prusim, Poland), whereas sodium dodecyl sulphate (SDS) and Sudan III were purchased from Merck Chemical (Saint Louis, MO, USA). All reagents were of the analytical grade.

### 2.2. Emulsions Preparation

The processing of FOC into Flaxseed Oil Cake Extract (FOCE) (used as a liquid phase) was carried out following the procedure described elsewhere [5]. Starch Capsul^®^ and maltodextrin were used as a wall material in the ratio 1:1 (8.5 g of each per 100 mL of FOCE). The wall components were added to the liquid phase at 25 °C and the mixture was stirred (250 rpm) until completely dissolved (approximately 15 min). FO was then added to the solution at a concentration of 10% and 20%, with respect to the total solids content of mixture. The model emulsions were prepared in two steps. First, the mixtures were mixed with FO for 5 min (500 rpm) using a magnetic stirrer (IKA, Staufen, Germany). Then, the mixtures were homogenized for 5 min at 1500 rpm (Magic LAB UTC, IKA, Staufen, Germany). The reference samples (used distilled water as a liquid phase), which served for comparison, were treated in the same way. The obtained variants are summarized in Table 1.

### 2.3. Spray Drying Protocol

Flaxseed oil emulsions powders were obtained by spray-drying, using a lab-scale spray dryer (Büchi B-290, Büchi Labortechnik AGT, Flawill, Switzerland). The drying air inlet temperature of 180 °C was chosen, based on the results described in previous study [12]. The air flow was 40 m^3^/h. The drying air outlet temperature was maintained at 55 ± 5 °C. Dried powders were collected in a collection vessel and stored in darkness at 4 °C. The yield of the spray drying process was calculated according to the Formula (1):

(1) $$\mathrm{Yield}\;\left(\%\right)=\frac{\mathrm{mas}\;\mathrm{of}\;\mathrm{powder}\;\left(g\right)}{\mathrm{dry}\;\mathrm{matter}\;\mathrm{of}\;\mathrm{emulsion}\;\left(g\right)}\times 100\%$$

### 2.4. Powders Characterization

#### 2.4.1. Solubility and Total Solid Content

Total solid content of the powders was analyzed based on the methodology of AOAC (Association of Official Agricultural Chemists) standard method (no. 968.11) [16]. To determine the solubility, 1 g of the spray dried powders (*W*_1_) were weighed into Falcon tubes (*W*_0_), to which 10 mL of distilled water was then added. Subsequently, the samples were centrifuged (700× *g*) for 2 min. The supernatants were decanted, and tubes were dried for 24 h at 50 °C. After drying, the tubes’ weight was determined again (*W*_2_). The solubility was calculated according to Equation (2) [17]:

(2) $$\mathrm{Solublity}=\frac{W_{2\;}-W_{0}}{W_{1}}\;\times 100$$

#### 2.4.2. Bulk and Tapped Densities

Bulk (ρ*_b_*) and tapped (ρ*_t_*) densities were evaluated with a slightly modified procedures of Jinapong et al. [18]. For the ρ*_b_* measurements, 5 g of each spray-dried powder sample was gently loaded into 25 mL glass cylinders and the filled volume was read. To determine ρ_t_, cylinders with powders used for the determination of ρ_b_ were tapped for 2 min using the automated tap density analyzer (Autotap, Quantachrome GmbH and Co. KG, Odelzhausen, Germany). The bulk and tapped densities were calculated according to the Formulas (3) and (4):

(3) $$\rho_{b}=\;\frac{\mathrm{powder}\;\mathrm{weight}\;\left(g\right)}{\mathrm{powder}\;\mathrm{volume}\;\mathrm{before}\;\mathrm{tapping}\;\left({\mathrm{cm}}^{3}\right)}$$

(4) $$\rho_{t}=\;\frac{\mathrm{powder}\;\mathrm{weight}\;\left(g\right)}{\mathrm{powder}\;\mathrm{volume}\;\mathrm{after}\;\mathrm{tapping}\;\left({\mathrm{cm}}^{3}\right)}$$

#### 2.4.3. Flowability and Cohesiveness of Powders

The flowability and cohesiveness of the powders were expressed using Carr’s index (*C*) and the Hausner ratio (*HR*), based on methodology proposed by Carr [19] and Hausner [20], respectively. The scale according to Reddy et al. (presented in Table 2) was used to characterize the powders’ flowability (*C*) and cohesiveness (*HR*) [21]. The indices were calculated from the bulk (ρ*_b_*) and tapped (ρ*_t_*) densities, according to the Formulas (5) and (6):

(5) $$C\left(\%\right)=\frac{\rho_{t}-\rho_{b}}{\rho_{t}}\times 100$$

(6) $$HR=\frac{\rho_{t}}{\rho_{b}}$$

### 2.5. Emulsions Reconstitution and Characterization

To obtain the reconstituted emulsions, the individual powders were added to distilled water to obtain a starting dry matter content in the emulsions, taking into account the total solids content of the powders, and were mixed for 15 min (200 rpm). All measurements were carried out for the initial (freshly prepared) and reconstituted emulsions, in triplicates.

#### 2.5.1. Determination of Particle Size Changes in the Emulsion Samples and Optical Microscope Observations of Emulsions

The particle size distribution of emulsions was performed using a Mastersizer 2000 (Malvern Instrument Ltd., Worcestershire, UK). The initial and reconstituted emulsion samples were gently diluted with 0.1% SDS solution, then dispersed in distilled water (stirred speed—2000 rpm) to obtain an obscuration rate of 10%. The optical properties of the sample were defined as follows—refractive index 1500 and absorption 1.00. Droplet size measurements were described as the volume-weighted mean diameter d_4,3_ = ∑n_i_d_4i_/∑n_i_d_i3_ and d_3,2_ = ∑n_i_d_3i_/∑n_i_d_i2_, where n_i_ is the number of droplets of diameter d_i_ [5].

Samples of initial and reconstituted emulsions were mixed in the ratio 1:1 with 0.5% SDS. The Sudan III was added as an oil dye to obtain a contrast. The initial and reconstituted emulsions were observed at 25 °C, using a digital camera connected to a microscope (OptaTech, Warsaw, Poland) at a magnification of ×10.

#### 2.5.2. Emulsions Stability

Emulsions stability was evaluated as the Emulsion Stability Index (ESI) and Creaming Index (*CI*), in the case of the initial and reconstructed emulsions. Immediately after the emulsions preparation, 25 mL of each emulsion were transferred to Egertz tubes and stored in refrigerator at 4 °C, and the volume of the upper phase was measured after 24, 48, and 168 h. The stability was measured by % of separation and expressed as Formula (7):

(7) $$CI\left(\%\right)=100-\left(\frac{H_{1}}{H_{0}}\;\times 100\right)$$

where *H*_0_ represents the emulsion initial height and *H*_1_ is the upper phase height [22,23].

To determine ESI, 20 µL of each emulsion were mixed with 5 mL of 0.1% SDS solution and vortexed. The absorbance was measured at 500 nm immediately and after 10 min (UV-VIS Evolution 220 spectrophotometer, Thermo-Scientific, Waltham, MA, USA). The stability of emulsions was expressed as ESI, based on the Equation (8):

(8) $$\mathrm{ESI}\left(\min\right)=\frac{A_{0}}{A_{0}-A_{10}}\times t$$

where *A*_0_ is the initial absorbance (0 min), *A*_10_ is the absorbance after 10 min, and t is the time between measurements (10 min) [24].

#### 2.5.3. Emulsion Color Measurements

The color of initial and reconstituted emulsions was determined by a Konica Minolta CR-5 colorimeter (Konica Minolta, Osaka, Japan). The values measured were L* (white 100/black 0), a* values (red positive/green negative), and b* values (yellow positive/blue negative). The Whiteness Index (WI), Yellowness Index (YI), and total color difference (*ΔE*) were calculated using the following Formulas (9)–(11) [11]:

(9) $$\mathrm{WI}=100-{\left[\left(100-L^{*}\right)+a^{2}+b^{2}\right]}^{0.5}$$

(10) $$\mathrm{YI}=142.86\times b\times L^{-1}$$

(11) $$\;\Delta E={\left[{\left(L_{\mathrm{standard}\;}-L_{\mathrm{sample}}\right)}^{2\;}+\;{\left(a_{\mathrm{standard}}-a_{\mathrm{sample}}\right)}^{2}+{\left(b_{\mathrm{standard}}-b_{\mathrm{sample}}\right)}^{2}\right]}^{0.5}$$

#### 2.5.4. Emulsions Rheological Measurements

The viscosity of the samples was analyzed using a rheometer (AR G2, TA Instruments Ltd., New Castle, DE, USA) with a stainless-steel cone plate geometry of 62 mm diameter and 1° cone angle. The steady-state flow procedure was performed in the range of 0.1 to 100 s^−1^, in triplicates at 20 °C. The TA Rheology Advantage Data Analysis V 5.4.7 software was used to record and analyze the rheological data. The Herschel-Bulkley model was applied to describe the rheological behavior of the initial and reconstituted emulsions as Formula (12):

(12) $$\tau =\tau_{0}+k{\dot{\gamma }}^{n}$$

where τ—the shear stress (Pa), τ_0_—the yield stress (Pa), $\dot{\gamma }$—the shear rate (s^−1^), *k*—the consistency index (Pa·s*^n^*), and *n*—the flow index.

### 2.6. Statistical Analyses

All results are presented as mean ± standard deviation. All data were subjected to a one-way analysis of variance (ANOVA) test using the Statistica 13.0 software (StatSoft, Kraków, Poland). Significant differences between means were determined by Fisher’s LSD (Least Significant Difference) NIR multiple comparison tests at *p* < 0.05. All experiments were replicated three times.

## 3. Results and Discussion

### 3.1. Powders Characteristics

The characteristics of spray-dried emulsions powders are shown in Table 3 and Table 4. One of the most pivotal physical parameters evaluated in case of powders are bulk and tapped densities. It was noticed that the application of FOCE as an emulsifying agent (and partially as a carrier) employed in the presented study was a factor that mostly caused the noticed differences for the bulk and tapped densities of the powders. The highest tapped density (0.432 ± 0.001 g/cm^3^) was observed for the powder, with 20% of FO containing FOCE (FOCE-MS-20%), in contrary to the lowest (0.347 ± 0.017 g/cm^3^), which was noticed for the sample without FOCE and the same content of oil (MS-20%) (*p <* 0.05). Similarly, this powder exhibited the lowest bulk density (0.306 ± 0.015 g/cm^3^). This observation indicated that FOCE could be a good carrier and emulsifying agent for spray-dried emulsions with a high oil content. The obtained result for FOCE-based powders were in contrast to the samples with distilled water used as the emulsion liquid phase, where a decrease of bulk and tapped densities with the increase of oil volume were observed (*p* < 0.05). Based on the bulk and tapped densities, the Carr index (*C*) and Hausner ratio (*HR*) were calculated. These parameters give an indication of the flowability and cohesiveness of the powders, respectively. Specifically, the Carr index describes the compressibility of a powder. The highest *C* and *HR* were observed for samples FOCE-MS-10% (*C-*25.54 ± 0.49 and *HR*-1.29 ± 0.01) and MS-20% (*C-*18.01 ± 0.78 and *HR*-1.23 ± 0.12). These results were comparable for the chitosan-based powders [25]. Based on the scale presented in Table 2, the best properties were exhibited by powder FOCE-MS-20% and was classified as “good”. One of the most reliable criterion to evaluate the behavior of powder in an aqueous solution is solubility. This process is attained after the powder undergoes dissolution and consists of the steps—sinkability, dispersibility, and wettability [17]. Additionally, a good solubility is important to obtain a redispersibility of spray-dried emulsion. The powder solubility is often correlated with the type of wall material. Maltodextrin is the one of the most popular polysaccharides used for purposes of the spray-drying microencapsulation. It is characterized by good solubility, and additionally low viscosity at high concentrations, and a neutral aroma and taste [25]. As shown in Table 4, all powders showed high solubility and a statistical important difference was observed only between samples FOCE-MS-10% and MS-20% (*p* < 0.05). In the case of sample MS-20%, it could be an effect of the higher concentration of maltodextrin in the dry matter of powder, and a lower viscosity of this emulsion. The highest yield was noted for sample MS-10% (57.98 ± 0.53%) and the lowest was observed for FOCE-MS-10% (44.26 ± 0.10%). According to Tonon et al., increasing carrier concentration decreases the process yield [26]. This might be an explanation for the decreased yield in this study, which is observed for samples with FOCE. The dry matter content of FOCE was reported at approximately 3% and consists of flaxseed proteins, polysaccharides (flaxseed gum), and another extractable compounds [5]. In the presented study, FOCE was used as a liquid phase and its components played the role of emulsifying agents, but should also be calculated for the total solids concentration. The addition of FOCE caused the higher total solids content in the initial emulsions and influenced the process yield, as shown in Table 4.

### 3.2. Emulsion Characteristics

#### 3.2.1. Emulsion Particles Size Changes

**Figure 1:**
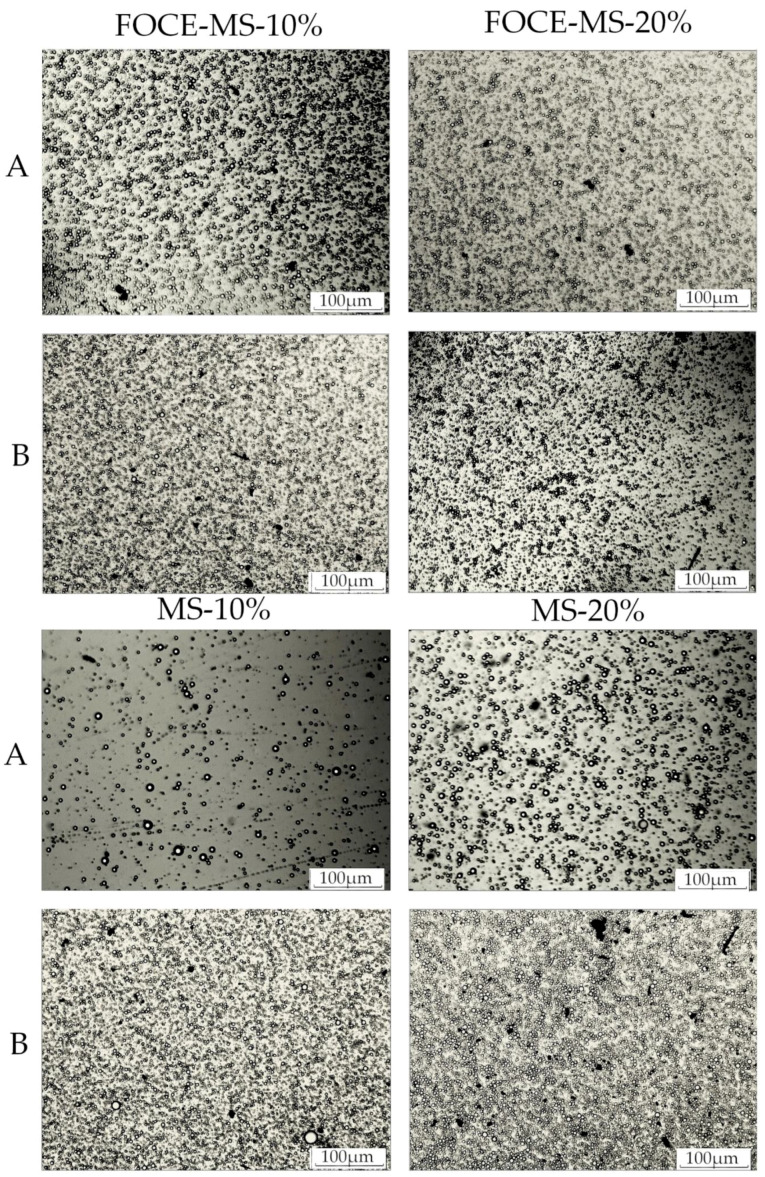
Optical microscopic images of emulsions (×10). A Samples before spray drying; and B samples after reconstitution. FOCE-MS-10%—FOCE-based emulsion with 10% of flaxseed oil; FOCE-MS-20%—FOCE-based emulsion with 20% of flaxseed oil; MS-10%—emulsion without FOCE with 10% of flaxseed oil; MS-20%—emulsion without FOCE with 20% of flaxseed oil.

The particles size distribution changes in the emulsion samples before and after reconstitution are presented in Table 5 and Figure 1. The droplet size was essential for the stability of the emulsion, as emulsions with a controlled particle size exhibited greater stability. Moreover, it was suggested that the smaller the droplets, the higher was the emulsion stability [5]. In fact, the lowest D_4,3_ (2.693 ± 0.00 µm) and D_3,2_ (2.166 ± 0.01 µm) after reconstitution were observed for the sample FOCE-MS-20%. As could be seen, the results of the emulsions stability were in line with the decreasing particle size distribution. It was observed that the surface area (D_3.2_) decreased after reconstitution in the FOCE-based emulsions (*p* < 0.05). On the contrary, for samples MS-10% and MS-20%, an increase was noticed (*p* < 0.05). The obtained results were supported by the captures presented in Figure 1. Emulsions with FOCE were more concentrated before spray drying, which presumably influenced their stability after reconstitution. Additionally, it could be observed that the spray drying process caused a higher concentration of emulsions in samples without FOCE. Sample MS-20% exhibited exceptionally low stability, as previously described. A tendency of agglomeration for the particles, before and after spray drying, was observed. These results were in line with the creaming index of the sample. In samples with FOCE, the observed distribution was similar before and after spray drying, therefore, it could also be concluded that the spray drying only decreased the particle size and surface area and did not cause a negative reaction such as flocculation or Ostwald ripening. According to Koç et al. [1], the increase of surface area was a negative phenomenon because the oil droplets might not be effectively covered by wall materials, and finally the stability of emulsions could be lower. Thus, the maltodextrin–starch mix used as a wall material should be additionally supported by some emulsifying agent for spray drying, and FOCE could be useful for this process, as demonstrated in the case of FOCE-based emulsions. The particle size in emulsions had a significant influence on their long-term stability, because the creaming velocity of an individual droplet was directly proportional to the square of its radius and to the density difference between the dispersed and the continuous phase [27]. It is recommended that the emulsion stability be evaluated with several indicators, because the stability effect is a synergy of oil binding, water holding, the rheological properties, and particle adhesion. According to Aizawa [28], turbidity measurements such as ESI are related to the size and number of oil droplets present in the emulsion. Thus, the variation in emulsion turbidity could indicate the changes of particles size and dispersity. In unstable emulsions, several changes could be observed, especially in the increasing particle size and concentrations, due to the coagulation and coalescence of particles. An observed increase in particle size of samples without FOCE, after the spray drying process could indicate emulsion stability disturbances.

#### 3.2.2. Emulsion Stability

**Figure 2:**
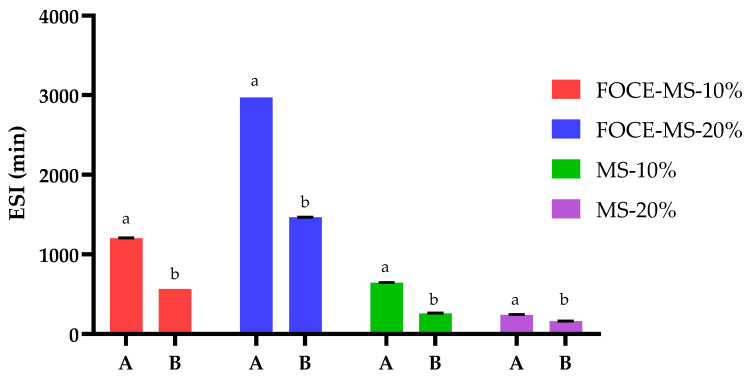
Emulsion Stability Index (ESI) of emulsions before spray drying A and after reconstitution B. FOCE-MS-10%—FOCE-based emulsion with 10% of flaxseed oil; FOCE-MS-20%—FOCE-based emulsion with 20% of flaxseed oil; MS-10%—emulsion without FOCE with 10% of flaxseed oil; MS-20%—emulsion without FOCE with 20% of flaxseed oil. Means with different lowercase are significantly different at *p* < 0.05.

**Figure 3:**
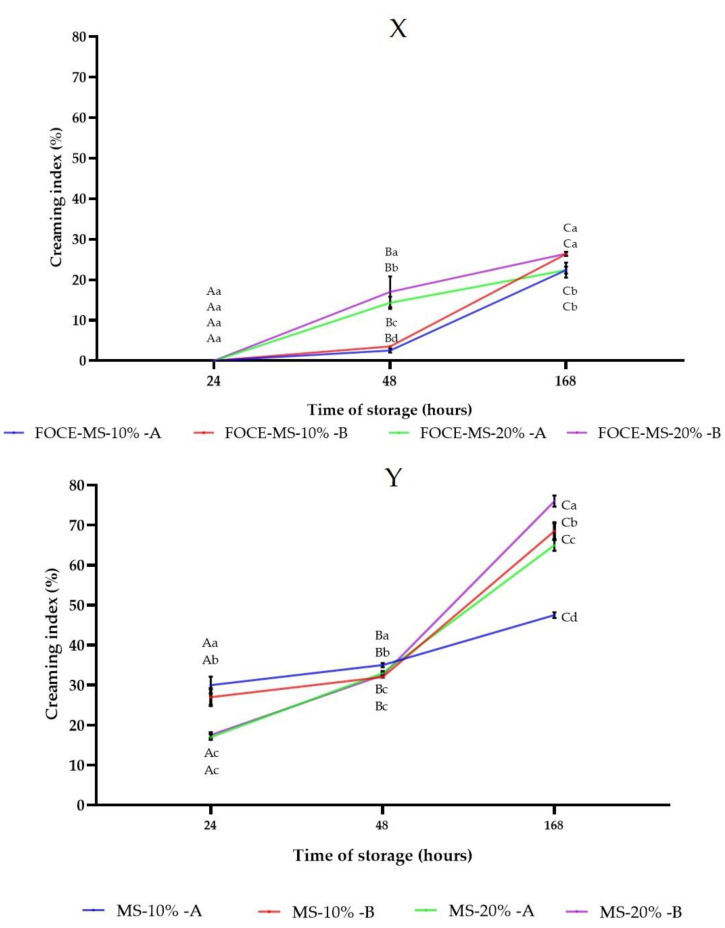
Creaming Index (*CI*) values of samples. (**X**) FOCE-based emulsions before spray drying A and after reconstitution B. Means with different lowercase are significantly different at *p* < 0.05. (**Y**) Emulsions without FOCE before spray drying A and after reconstitution B. Means with different letters are significantly different at *p <* 0.05.

Figure 2 and Figure 3 present the stability of the initial (A) and reconstituted (B) emulsions, expressed as ESI and CI. The emulsions based on FOCE showed significantly higher ESI than the emulsions where distilled water was used as the liquid phase (*p* < 0.05). However, for all samples, a significant decrease of ESI after reconstitution was noticed (*p <* 0.05). The highest ESI was noticed for the initial FOCE-MS-20% sample (2970.00 ± 0.00 min). Moreover, it could be observed that emulsions with FOCE showed a significantly higher ESI in case of samples with a 20% content of oil (A—2970.00 ± 0.00 min; B—1465.50 ± 0.50 min) than with 10% (A—1201.25 ± 0.52 min; B—566.67 ± 0.00 min) (*p* < 0.05). These results were opposite to samples without FOCE. Emulsions with distilled water as a liquid phase represented lower ESI values when the oil fraction increased. Maltodextrin and starch exhibited emulsifying properties, but these mechanisms were quite different than that for the protein and polysaccharides included in FOCE. As previously reported, the FOCE emulsifying activity is a result of synergistic action of its two main fractions—protein and gum [5]. Flaxseed protein adsorbs onto the oil–water surface over the emulsification process. Due to the presence of polysaccharides such as flaxseed gum steric and mechanical effects, the emulsion system could be stabilized by the highly branched polysaccharide structure. Additionally, flaxseed gum and flaxseed protein have the ability to reduce the surface tension [25,26,29]. Presumably maltodextrin and starch influenced the stability of emulsions, by increasing the viscosity and ability to gelation of the aqueous continuous phase surrounding the oil droplets [3,18]. Thus, the use of various combinations of maltodextrin and other compounds such as proteins and polysaccharides for the purpose of microencapsulation is suggested [4]. Modified starches exhibited the ability of formation of less porous materials [30]. Starches such as OSA or Capsul^®^ were selected as wall materials, due to their amphiphilic nature and the consequent biosafety of their derivatives [31]. Capsul^®^ used in this study was a chemically modified starch, produced by incorporating a lipophilic component (using octenyl succinic anhydride), aimed at conferring emulsifying properties [32]. As a result of this modification, hydrophilic starch gains hydrophobic octenyl groups, resulting in molecules of an amphiphilic nature [29].

The aim of this modification was giving the material a capacity for retaining volatiles during atomization in a spray-dryer [32].

As shown in Figure 3, the emulsions without FOCE represented higher *CI* and started creaming after 24 h (*p <* 0.05). Moreover, for these samples, the creaming index increased with an increased oil volume. These emulsions (initial and reconstituted) exhibited very high differences after 168 h, especially in the case of a sample with 10% oil (A—47.50 ± 0.71% and B—76.00 ± 1.41%). On the other hand, the creaming of samples with FOCE started after 48 h (*p* < 0.05). In the case of samples with FOCE after 24 h, the creaming index was 0% and the sample was stable (*p >* 0.05). Based on this observation, it is reasonable to conclude that FOCE has the ability to prevent emulsion creaming during storage, both before and after reconstitution from spray-dried powders.

Creaming is one of the most common mechanism of emulsion instability, causing macroscopic phase separation into cream and serum layers [29]. Creaming index provides information about the extent and viscosity of droplet aggregation in an emulsion. Emulsions with a low creaming index and a low creaming rate present a good creaming behavior, resulting in a good emulsion stability. Additionally, the *CI* rate influences a rheological behavior and is an indicator of the quality of the emulsion [33]. It is very important to choose an accurate ration of the wall material, especially in the case of maltodextrin. Rapid creaming could be an effect of exceeding a critical maltodextrin concentration. Many authors suggest that the high concentration of maltodextrin might cause the flocculation of droplets by unabsorbed maltodextrin molecules in the aqueous phase of the emulsion [30,31,32]. Klinkesorn et al. [23] observed that non-adsorbed polymers generate an osmotic force between droplets, which increases with growing polymer concentration. Finally, it becomes large enough to overcome the repulsive forces acting between the droplets. The better creaming stability of FOCE-based emulsions could be explained as the ratio of bound biopolymer concentration and type. According to Chanamai and McClements [34], the increase of biopolymers such as starch or maltodextrin in the aqueous phase caused higher viscosity and slowed down the upward movements in emulsions.

#### 3.2.3. Color of Emulsions

Table 6 summarizes the results of color measurements of emulsions before spray drying and after reconstruction. It could be observed that emulsions without FOCE exhibited higher differences of lightness (L*) as compared to the FOCE-based emulsions (*p* < 0.05). A similar tendency was noticed in the case of whiteness and yellowness indices (*p* < 0.05). The high decrease of lightness in the case samples without FOCE, might be caused by aggregation and destabilization [27,35]. It is known that the color of an oil-in-water emulsion is changed appreciably over time, due to the droplet growth caused by Ostwald ripening [27]. Changes of lightness were in line with the results for ESI and CI. The highest L* decrease was observed for the samples MS-10% and MS-20% and these emulsions exhibited the lowest ESI and the highest differences in creaming indices during storage, between variants A and B. In the case of the sample with FOCE, the lower initial lightness was affected by many factors. Flaxseed oil and FOCE exhibited more yellowness, as previously reported [5]. Additionally, maltodextrin is bright white in natural form and could exhibit higher lightness than FOCE [36]. According to Câmara et al. [37], in the case of more concentrated emulsions, a significant part of the fraction of the incident light could be propagated back to the surface. The observed increase of the b* value in the reconstituted emulsions might be linked to the Maillard reactions during the spray drying process [12]. This observation was in line with results reported for the spray-dried FOCE powders [12]. Moreover, a similar effect was observed by Verruck et al. [38] for goat milk spray dried with inulin. Indeed, a significant increase of the yellowness index of all samples after reconstitution was observed (*p* < 0.05). In contrast, Pham et al. [39] suggested that b* increase might be an effect of the formation of phenolics–protein complexes. The ∆E of each samples was higher than 1 and the statistical differences between samples were noticed (*p* < 0.05). The highest ∆E (14.68 ± 0.01) was observed for the sample MS-10% and the lowest was noticed for FOCE-MS-20% (1.61 ± 0.07). When ∆E was higher than 1, the difference was considered perceptible to the human eye [12]. This parameter was a visualization of all changes in sample. Significantly lower values of ∆E were observed for the FOCE-based reconstituted emulsions, as compared to the initial emulsions with water as the liquid phase. Gu et al. [4] suggested that, in addition to color, emulsions formed by combining protein and polysaccharides are highly stable and resistant to a broad range of environmental stresses. The stress factor in the present study was the spray drying and reconstitution process. Thus, it is reasonable to conclude that FOCE not only played the role of emulsifier, but additionally prevented emulsion destabilization caused by stress factors.

#### 3.2.4. Emulsion Rheological Characteristics

**Figure 4:**
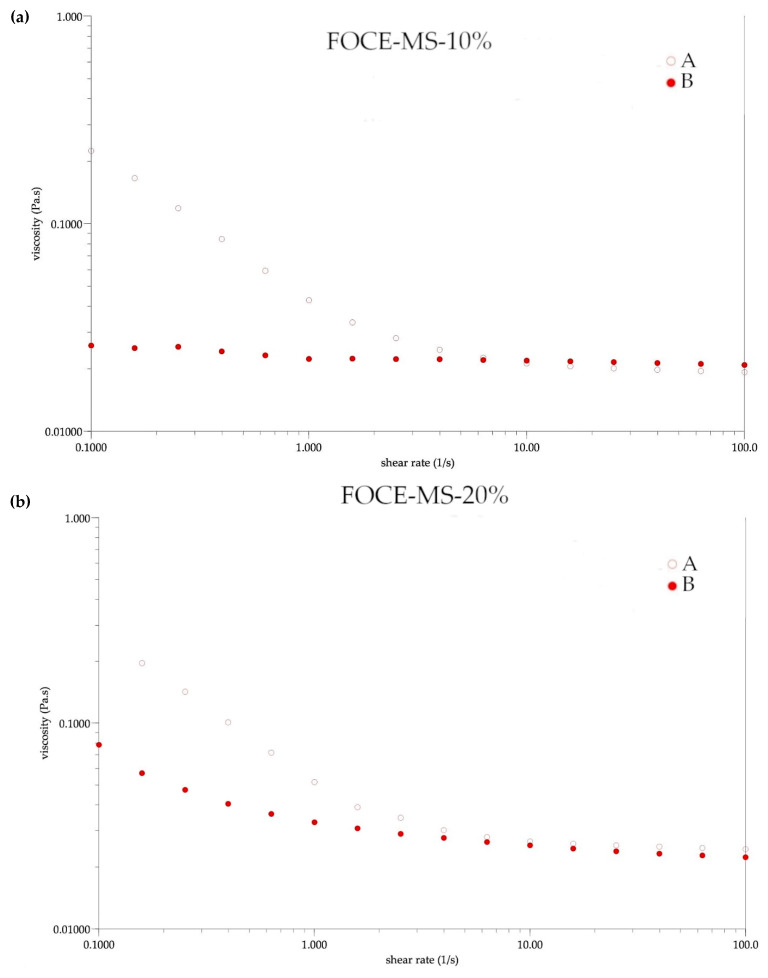
The flow curves of emulsions with FOCE before spray drying A and after reconstitution B. (**a**) FOCE-MS-10%—FOCE-based emulsion with 10% of flaxseed oil; (**b**) FOCE-MS-20%—FOCE-based emulsion with 20% of flaxseed oil.

**Figure 5:**
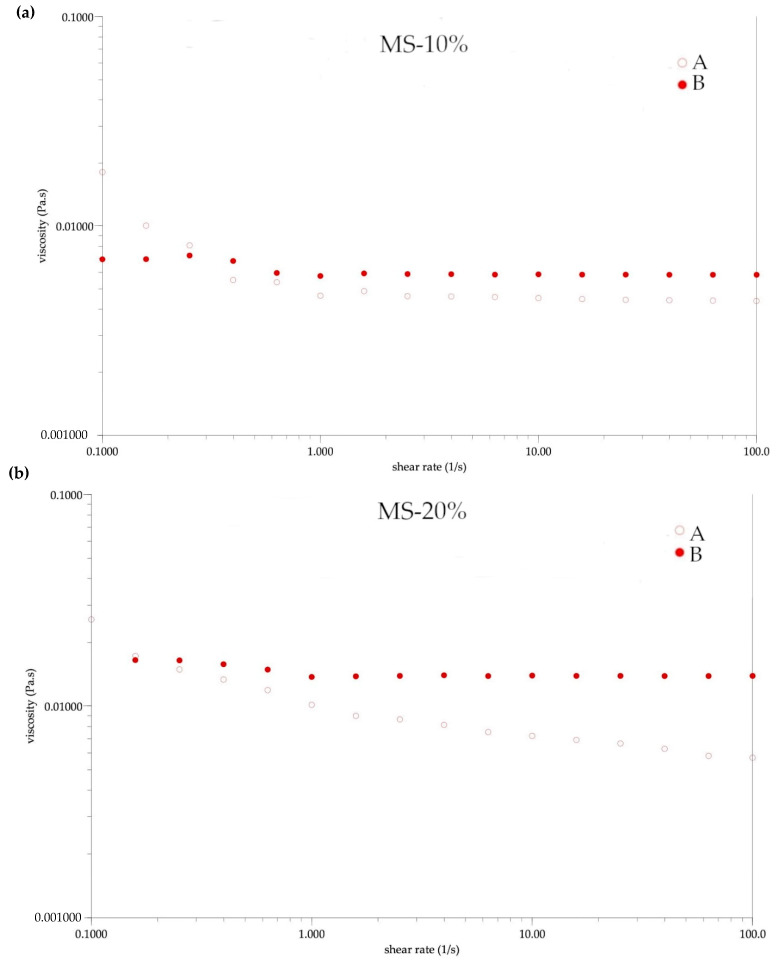
The flow curves of emulsions without FOCE before spray drying A and after reconstitution B. (**a**) MS-10%—emulsion without FOCE with 10% of flaxseed oil; (**b**) MS-20%—emulsion without FOCE with 20% of flaxseed oil.

The rheological parameters of the initial (A) and reconstituted emulsions (B) based on the Hershel–Bulkey model are presented in Table 7, and their flow curves are illustrated in Figure 4 and Figure 5. Based on the flow curves and n value a mixed rheological character of the emulsions was noticed. The n less than 1 indicated that the emulsion exhibited Newtonian character [22]. In the case of the initial FOCE-MS-10%, MS-10%, and MS-20%, a non-Newtonian character of the samples was observed. It could be observed that these samples exhibited an n value indicated by a nearly Newtonian character, after the reconstitution process. A similar flow behavior was reported for the flaxseed oil-in-water emulsions stabilized with whey protein isolate (WPI) [40], as well as for avocado oil-in-water emulsions stabilized by WPI/maltodextrin [41]. Based on the results of the emulsions flow behavior after reconstitution, it could be concluded that all samples had a nearly shear thinning behavior. A similar observation was reported by Kumar et al. in case of emulsions with chitozan as a wall material [25]. After the spray drying process, each emulsion exhibited a lower yield stress value and a lower viscosity (*p* < 0.05), only sample MS-20% was an exception. The viscosity of the initial emulsions decreased with the increase of oil volume. The same observations were made by Kurek and Pratap-Singh in case of emulsions with various plant protein (hemp, pea, and rice), in combination with maltodextrin [42]. Additionally, this trend was observed by Di Giorgio et al. [43] for emulsions with soya protein, which are often compared to flaxseed protein due to their similarity [13]. The observed higher viscosity of the samples with FOCE after reconstitution, was in line with this effect described by Boonalao et al. [44], in the case of xanthan gum. The addition of polysaccharides such as xanthan gum, which is often compared to flaxseed gum, increases the viscosity of aqueous solutions, enhancing their stability, because it prevents the movement of droplets, which causes creaming. In terms of consistency index, the samples with FOCE indicated the tendency of an increase in K with an increase in oil volume, which was contrary to the samples without FOCE (*p* < 0.05). Results obtained for the MS-10% and MS-20% samples were in agreement with the results reported by Kumar et al. [25], who observed that the consistency index had a lower value in emulsions (stabilized by hemp, rice, and pea protein), with a 20% oil content, than in emulsions with a lower oil content.

## 4. Conclusions

This study concluded that FOCE might be applied to stabilize spray-dried flaxseed oil-in-water reconstructable emulsions, which are not described so far. Despite the ability of starch and maltodextrin for emulsion stabilization, these mechanisms were insufficient to obtain long-term stable emulsions. Thus, maltodextrin and starch could have only played the role of wall material. FOCE could be an adequate emulsifier for the spray-dried emulsions, with a high oil content providing their high stability, after reconstitution, when compared to emulsions based only on a maltodextrin–starch wall material, with water as the liquid phase. Based on our findings, it is reasonable to conclude that FOCE could be an alternative bio-based emulsifier used for microencapsulated functional foods. Furthermore, powdered flaxseed oil emulsions with FOCE could also be applied as a raw material during the production of processed food and as fat bases for the dairy and meat industries. Moreover, there is a potential for these products to be used in pharmaceutical, nutraceutical, and cosmetical applications, where nature-based solutions are desired. The proposed solution fits the rules of the circular economy and the idea of zero-waste.

## Figures and Tables

**Table 1:** Composition of the model emulsions.

Sample Name	Maltodextrin: Starch Ratio	Liquid Phase	Oil Content *
FOCE-MS-10%	1:1	FOCE	10%
FOCE-MS-20%	1:1	FOCE	20%
MS-10%	1:1	Distilled Water	10%
MS-20%	1:1	Distilled Water	20%

* The total solids concentration refers to the (wall material + oil) ratios in the emulsion calculated and represented on a dry basis. FOCE—flaxseed oil cake extract, MS—maltodextrin: starch.

**Table 2:** Specifications for the Carr’s index and the Hausner ratio.

	Carr’s Index	Hausner Ratio
Excellent	0–10%	1.00–1.11
Good	10–15%	1.12–1.18
Fair	16–20%	1.19–1.25
Possible	21–25%	1.26–1.34
Poor	26–31%	1.35–1.45
Very poor	32–37%	1.46–1.59
Very very poor	>38%	>1.60

**Table 3:** Bulk density, tapped density, Hausner ratio (*HR*), and Carr index (*C*) of the spray-dried emulsion powders.

Powder Sample	$\rho_{b}\;(g/{\mathrm{cm}}^{3})$	$\rho_{t}\;(g/{\mathrm{cm}}^{3})$	*HR*	*C* (%)
FOCE-MS-10%	0.313 ± 0.001 ^b^	0.405 ± 0.004 ^c^	1.29 ± 0.01 ^a^	25.54 ± 0.49 ^a^
FOCE-MS-20%	0.370 ± 0.001 ^a^	0.432 ± 0.001 ^a^	1.17 ± 0.00 ^d^	14.29 ± 0.00 ^d^
MS-10%	0.351 ± 0.018 ^a^	0.424 ± 0.001 ^b^	1.21 ± 0.06 ^c^	17.14 ± 0.41 ^c^
MS-20%	0.306 ± 0.015 ^b^	0.347 ± 0.017 ^d^	1.23 ± 0.12 ^b^	18.01 ± 0.78 ^b^

Values are means ± standard deviation of the triplicate determinations. Means with different letters in the same column are significantly different at *p* < 0.05. $\rho_{b}$—bulk density;${\;\rho }_{t}$—tapped density; *HR*—Hausner ratio; *C*—Carr index; FOCE—flaxseed oil cake extract; MS—maltodextrin: starch.

**Table 4:** Solubility, yield, and total solids content (TSC) of the spray-dried emulsion powders.

Powder Sample	Solubility (%)	Yield (%)	TSC (%)
FOCE-MS-10%	94.71 ± 2.47 ^a^	44.26 ± 0.10 ^c^	98.11 ± 0.57 ^a^
FOCE-MS-20%	98.71 ± 1.87 ^ab^	46.58 ± 0.82 ^d^	98.68 ± 1.00 ^a^
MS-10%	98.61 ± 1.05 ^ab^	57.98 ± 0.53 ^a^	95.15 ± 0.15 ^b^
MS-20%	96.67 ± 2.00 ^b^	54.98 ± 0.42 ^b^	96.76 ± 1.00 ^b^

Values are means ± standard deviation of triplicate determinations. Means with different letters in the same column are significantly different at *p* < 0.05. FOCE—flaxseed oil cake extract; MS—maltodextrin: starch.

**Table 5:** Particle size distribution of samples.

Sample	FOCE-MS-10%	FOCE-MS-20%	MS-10%	MS-20%
D_4.3_ (µm)				
A	3.225 ± 0.05 ^Aa^	2.700 ± 0.01 ^Ba^	3.206 ± 0.12 ^Cb^	3.045 ± 0.00 ^Db^
B	3.065 ± 0.01 ^Ab^	2.693 ± 0.00 ^Bb^	3.342 ± 0.00 ^Ca^	3.069 ± 0.01 ^Da^
D_3.2_ (µm)				
A	2.327 ± 0.02 ^Aa^	2.445 ± 0.02 ^Ba^	2.206 ± 0.05 ^Cb^	2.256 ± 0.01 ^Db^
B	2.271 ± 0.02 ^Ab^	2.166 ± 0.01 ^Bb^	2.280 ± 0.00 ^Ca^	2.305 ± 0.00 ^Da^

A Samples before spray drying; and B samples after reconstitution. Values are means ± standard deviation of triplicate determinations. Means with different lowercase in the same column are significantly different at *p <* 0.05. Means with different uppercase in the same raw are significantly different at *p <* 0.05. FOCE—flaxseed oil cake extract; MS—maltodextrin: starch; FOCE-MS-10%—FOCE-based emulsion with 10% of flaxseed oil; FOCE-MS-20%—FOCE-based emulsion with 20% of flaxseed oil; MS-10%—emulsion without FOCE with 10% of flaxseed oil; MS-20%—emulsion without FOCE with 20% of flaxseed oil; D_4.3_—volume-weighted mean diameter; D_3.2_—volume/surface mean diameter.

**Table 6:** Color of emulsions before spray drying (A) and after reconstitution (B).

Sample	FOCE-MS-10%	FOCE-MS-20%	MS-10%	MS-20%
L*				
A	86.99 ± 0.00 ^Aa^	87.07 ± 0.00 ^Aa^	97.06 ± 0.00 ^Ca^	96.69 ± 0.00 ^Da^
B	84.42 ± 0.01 ^Ab^	86.92 ± 0.01 ^Bb^	83.28 ± 0.00 ^Cb^	87.70 ± 0.00 ^Db^
a*				
A	−1.83 ± 0.01 ^Aa^	−1.06 ± 0.01 ^Ba^	−0.17 ± 0.01 ^Ca^	−0.07 ± 0.01 ^Da^
B	−2.33 ± 0.01 ^Ab^	−1.64 ± 0.01 ^Bb^	−1.81 ± 0.01 ^Cb^	−1.18 ± 0.01 ^Db^
b*				
A	16.92 ± 0.01 ^Ab^	15.88 ± 0.02 ^Bb^	4.32 ± 0.02 ^Cb^	5.48 ± 0.02 ^Db^
B	19.91 ± 0.02 ^Aa^	17.37 ± 0.01 ^Ba^	9.08 ± 0.02 ^Ca^	15.54 ± 0.02 ^Da^
YI				
A	28.63 ± 0.01 ^Ab^	26.06 ± 0.12 ^Bb^	6.36 ± 0.01 ^Cb^	8.10 ± 0.01 ^Db^
B	32.70 ± 0.04 ^Aa^	28.55 ± 0.01 ^Ba^	15.58 ± 0.01 ^Ca^	25.31 ± 0.01 ^Da^
WI				
A	76.14 ± 0.02 ^Ab^	79.49 ± 0.02 ^Ba^	94.77 ± 0.00 ^Ca^	93.59 ± 0.00 ^Da^
B	76.88 ± 0.00 ^Aa^	78.19 ± 0.00 ^Bb^	80.88 ± 0.00 ^Cb^	80.15 ± 0.00 ^Db^
ΔE				
A	Used as standard	Used as standard	Used as standard	Used as standard
B	3.98 ± 0.01 ^A^	1.61 ± 0.07 ^B^	14.68 ± 0.01 ^C^	13.53 ± 0.01 ^D^

FOCE-MS-10%—FOCE-based emulsion with 10% of flaxseed oil; FOCE-MS-20%—FOCE-based emulsion with 20% of flaxseed oil; MS-10%—emulsion without FOCE with 10% of flaxseed oil; MS-20%—emulsion without FOCE with 20% of flaxseed oil; L*—lightness; a*—redness/greenness; b*—yellowness/blueness; YI—yellowness index; WI—whiteness index; ΔE—total color difference. Values are means ± standard deviation of triplicate determinations. Means with different lowercase in the same column are significantly different at *p <* 0.05. Means with different uppercase in the same raw are significantly different at *p <* 0.05.

**Table 7:** Rheological parameters of emulsions before spray drying (A) and after reconstitution (B), based on Hershel–Bulkey.

Sample	FOCE-MS-10%	FOCE-MS-20%	MS-10%	MS-20%
τ_y_ (Pa)				
A	0.0534 ± 0.000 ^Aa^	0.0520 ± 0.001 ^Ba^	0.0090 ± 0.003 ^Ca^	0.0026 ± 0.004 ^Da^
B	0.0225 ± 0.001 ^Ab^	0.0255 ± 0.000 ^Bb^	0.0030 ± 0.002 ^Cb^	0.0032 ± 0.002 ^Ca^
Viscosity (Pa·s)				
A	0.2710 ± 0.001 ^Aa^	0.2350 ± 0.001 ^Ba^	0.0450 ± 0.000 ^Ca^	0.0130 ± 0.001 ^Da^
B	0.0197 ± 0.002 ^Ab^	0.0248 ± 0.000 ^Bb^	0.0057 ± 0.001 ^Cb^	0.0145 ± 0.000 ^Db^
*k* (Pa·s*^n^*)				
A	0.1638 ± 0.005 ^Aa^	0.3310 ± 0.010 ^Ba^	0.0150 ± 0.002 ^Ca^	0.0141 ± 0.001 ^Da^
B	0.0204 ± 0.001 ^Ab^	0.0263 ± 0.005 ^Bb^	0.0057 ± 0.001 ^Cb^	0.0138 ± 0.003 ^Db^
*n* (–)				
A	0.48 ± 0.02 ^Ab^	0.95 ± 0.01 ^Bb^	0.78 ± 0.00 ^Cb^	0.85 ± 0.02 ^Db^
B	1.00 ± 0.00 ^Aa^	0.98 ± 0.02 ^Ba^	1.00 ± 0.00 ^Aa^	1.00 ± 0.00 ^Aa^

FOCE-MS-10%—FOCE-based emulsion with 10% of flaxseed oil; FOCE-MS-20%—FOCE-based emulsion with 20% of flaxseed oil; MS-10%—emulsion without FOCE with 10% of flaxseed oil; MS-20%—emulsion without FOCE with 20% of flaxseed oil; τ_y_—yield stress; *k*—consistency index; *n*—flow index. Values are means ± standard deviation of triplicate determinations. Means with different lowercase in the same column are significantly different at *p <* 0.05. Means with different uppercase in the same raw are significantly different at *p <* 0.05.

## Data Availability

The data presented in this study are available on request from the corresponding authors.
